# Enhanced Deep-Learning Model for Carbon Footprints of Chemicals

**DOI:** 10.1021/acssuschemeng.3c07038

**Published:** 2024-02-05

**Authors:** Dachuan Zhang, Zhanyun Wang, Christopher Oberschelp, Eric Bradford, Stefanie Hellweg

**Affiliations:** † National Centre of Competence in Research (NCCR) Catalysis, Ecological Systems Design, Institute of Environmental Engineering, ETH Zürich, Zürich 8093, Switzerland; ‡ Technology and Society Laboratory, Empa-Swiss Federal Laboratories for Materials Science and Technology, St. Gallen CH-9014, Switzerland

**Keywords:** life cycle assessment, sustainable chemistry, product carbon footprint, machine learning

## Abstract

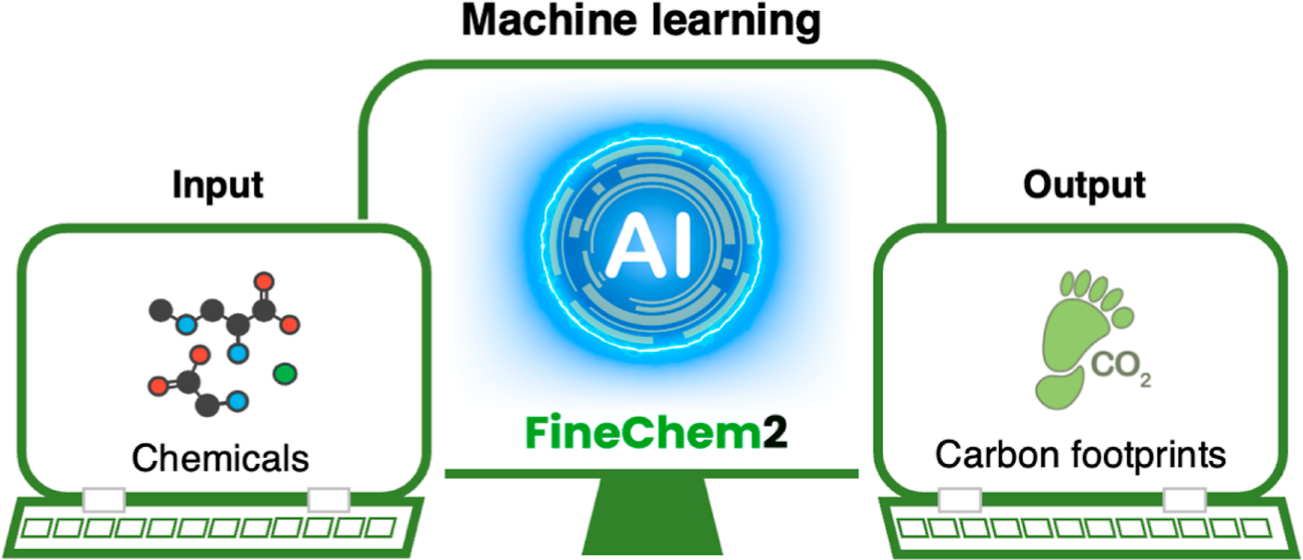

Millions of chemicals have been designed; however, their product carbon footprints (PCFs) are largely unknown, leaving questions about their sustainability. This general lack of PCF data is because the data needed for comprehensive environmental analyses are typically not available in the early molecular design stages. Several predictive tools have been developed to estimate the PCF of chemicals, which are applicable to only a narrow range of common chemicals and have limited predictive ability. Here, we propose FineChem 2, which is based on a novel transformer framework and first-hand industry data, for accurately predicting the PCF of chemicals. Compared to previous tools, FineChem 2 demonstrates significantly better predictive power, and its applicability domains are improved by ∼75% on a diverse set of chemicals on the global market, including the high-production-volume chemicals identified by regulators, daily chemicals, and chemical additives in food and plastics. In addition, through better interpretability from the attention mechanism, FineChem 2 may successfully identify PCF-intensive substructures and critical raw materials of chemicals, providing insights into the design of more sustainable molecules and processes. Therefore, we highlight FineChem 2 for estimating the PCF of chemicals, contributing to advancements in the sustainable transition of the global chemical industry.

## Abstract 2

Enhanced deep-learning model for estimating carbon footprints of chemicals with better accuracy, expansive applicability domain, and good interpretability.

## 1 Introduction

Chemicals are present in more than 90% of manufactured goods and, thus, influence the environmental impacts of nearly all sectors.^1^ In 2020, direct greenhouse gas (GHG) emissions from the petrochemical sector, including energy supply, amounted to 1.8 Gt CO_2_-equiv, equivalent to 4% of the global GHG emissions, and indirect GHG emissions from other industrial activities supplying inputs to the petrochemical industry accounted for another 6%.^2^ As the production capacity of the global chemical industry is expected to reach nearly double that in 2017 by 2030, a timely sustainability transition is crucial.^3^

To date, more than 250 million chemical substances have been designed and registered in the Chemical Abstracts Service database,^4^ among which over 300,000 have been industrialized.^5^ Increasing efforts have been devoted to the design of new, more sustainable chemicals to accelerate the sustainability transition of the chemical industry.^6^ Among others, life cycle assessment (LCA) has increasingly become a key method for evaluating the environmental impacts of chemical products and processes.^7^

However, the implementation of LCA in the chemical industry has mostly been restricted to case studies of already existing products and processes, with a particular focus on basic chemicals,^8^ hindering the overall decarbonization efforts of the chemical industry. This is owing to the fact that detailed life cycle inventory data for LCA are usually not available due to confidentiality issues or at the early design stage. Meanwhile, the optimal time to minimize the environmental burden of chemical production is in the early design stages of molecules and synthesis routes. After the synthesis routes are implemented, improvements are significantly more expensive and time-consuming to implement.^9^ In addition, because data on many chemicals are scarce, sustainability studies usually neglect or only crudely estimate the impacts of chemicals in final products (e.g., chemical additives in food and plastics), which affects the accuracy of LCA outcomes.^10,11^ Therefore, accurately calculating the product carbon footprint (PCF) of chemicals remains a major challenge in achieving more sustainable chemistry and products.

Against this backdrop, several predictive LCA (pre-LCA) tools were developed to fill this gap, for example, based on similarities between characterized and noncharacterized processes,^12^ molecular structures,^13^ process design and simulation,^14^ or a hybrid approach.^9^ Among them, the molecular structure-based approach has received the most attention as a screening tool thanks to its simplicity and low requirements for input data. In 2009, the first molecular structure-based pre-LCA tool, FineChem^13^ was developed based on artificial neural networks (ANNs) and basic molecular descriptors to estimate the environmental impact of chemicals. Subsequently, several other pre-LCA tools were proposed based on machine learning (ML) algorithms and mostly data from ecoinvent.^15−20^ For example, Sun et al. developed a new ANN model based on data processing strategies (ANN-DP) to enhance the predictive ability of the model.^15^ Song et al. trained multilayer ANNs to rapidly estimate the life cycle impacts of chemicals (rapid-ANN) and highlighted the importance of understanding the applicability domain (AD) of models.^16^ Calvo-Serrano et al. took molecular descriptors, thermodynamic properties, and σ-profiles as ML models’ input to predict the life cycle impacts of chemicals and demonstrated that using additional thermodynamic descriptors could improve the model performance.^17,18^ Kleinekorte et al. integrated molecular and process descriptors to enable ML models to distinguish the impacts of chemicals produced by different processes.^19^ Zhu et al. developed an ANN model for screening green chemical substitutes to replace trifluoroacetic anhydride, a chemical used in the sitagliptin production process.^20^

Despite their successful application in approximate PCFs of some chemicals, these existing models exhibit low accuracy and generative ability caused by the conventional ML algorithms and narrow AD because of limited training data, which restricts their application to basic chemicals with simple molecular structures. In addition, lower-quality proxy data related to chemical production has been widely used in ecoinvent, leading to errors in the PCF calculation.^21^ The contamination of proxy data is transmitted to these pre-LCA tools, compromising their performance. Furthermore, while ML has been successfully applied in many fields, interpretability remains challenging for ML-based models.^22,23^ Previous tools used Shapley additive explanations (SHAP)^24^ to identify the critical molecular descriptors that are relevant to PCFs. However, the detailed correlations between PCFs and functional groups and substructures cannot be distinctly quantified, which limits their application in the design of more sustainable molecules. Therefore, a robust tool with better accuracy, expansive AD, and better interpretability is required to determine the PCF of all chemicals.

In this study, we aim to overcome the limitations of low accuracy and limited applicability of previous pre-LCA tools by designing FineChem 2, a new tool built on high-quality chemical production data sets and the state-of-the-art transformer framework. We perform extensive evaluations on different testing data sets to validate the accuracy and robustness of FineChem 2. Based on our findings, we anticipate that FineChem 2 will serve as a useful tool for filling data gaps in guiding the design of sustainable molecules and production processes.

## 2 Methods and Materials

### 2.1 Data Set Construction

Deep learning relies on high-quality data for accurate predictions. To obtain a comprehensive data set on the PCF of chemicals, chemical production data sets from IDEA v2.3^25^ and ecoinvent v3.8^26^ were integrated, together with first-hand data from the industry. Noting that inappropriate proxy data are commonly used within the ecoinvent database to fill data gaps and significantly affect the data quality,^21^ an additional quality check was performed. More specifically, the ecoinvent data sets that are contaminated with direct proxy data or with major proxy use in their educts were excluded.

The molecular structures of the chemicals were retrieved from public databases, e.g., PubChem^27^ and ChEMBL,^28^ and stored in the simplified molecular input line entry system (SMILES) format. Subsequently, any chemicals below 80% purity or with metal ions, mixtures, polymers, and inorganic chemicals were removed from the data set. To demonstrate the diversity of the training data set, the number of unique molecular scaffolds of chemicals in different data sets was calculated according to the definition of the Murcko scaffold.^29^

The PCF of the chemicals was calculated based on the indicator of global warming, 100a, Intergovernmental Panel on Climate Change (IPCC) 2013^30^ using SimaPro.^31^ For the IDEA and ecoinvent data sets, the carbon footprint was adjusted by integrating the corresponding cradle-to-gate data sets from extraction of raw materials (like oil or natural gas) to the manufactured chemical product (e.g., ethylene). This includes the energy-related carbon footprint contributions, which were obtained by calculating the cradle-to-gate, cumulative electricity, and heat inputs along the chemical manufacturing supply chains. Data sets across data sets and data sources were harmonized by using the average chemical industry electricity and heat carbon footprints. To ensure consistency with the aggregated system process data sets in the supply chains of some chemicals in ecoinvent, the electricity and heat carbon footprints were based on the European chemical industry data. For electricity, the cradle-to-gate carbon footprint was 0.16 kg CO_2_-equiv per MJ electricity, whereas for heat, it was 0.071 kg CO_2_-equiv per MJ heat,^32^ to avoid biases due to individual modeling choices and to align with the European black box data sets prevalent in the chemical supply chains in ecoinvent. For multichemical production processes, ecoinvent used economic allocation to allocate impacts, and IDEA and the industry data sets used mass allocation. Data sets of solutions were adjusted to 100% active ingredients. For chemicals that are presented in more than one data source or presented more than once in one data source because of different production processes, their PCF values were averaged.

### 2.2 Construction of the FineChem 2 Model

**Figure 1:**
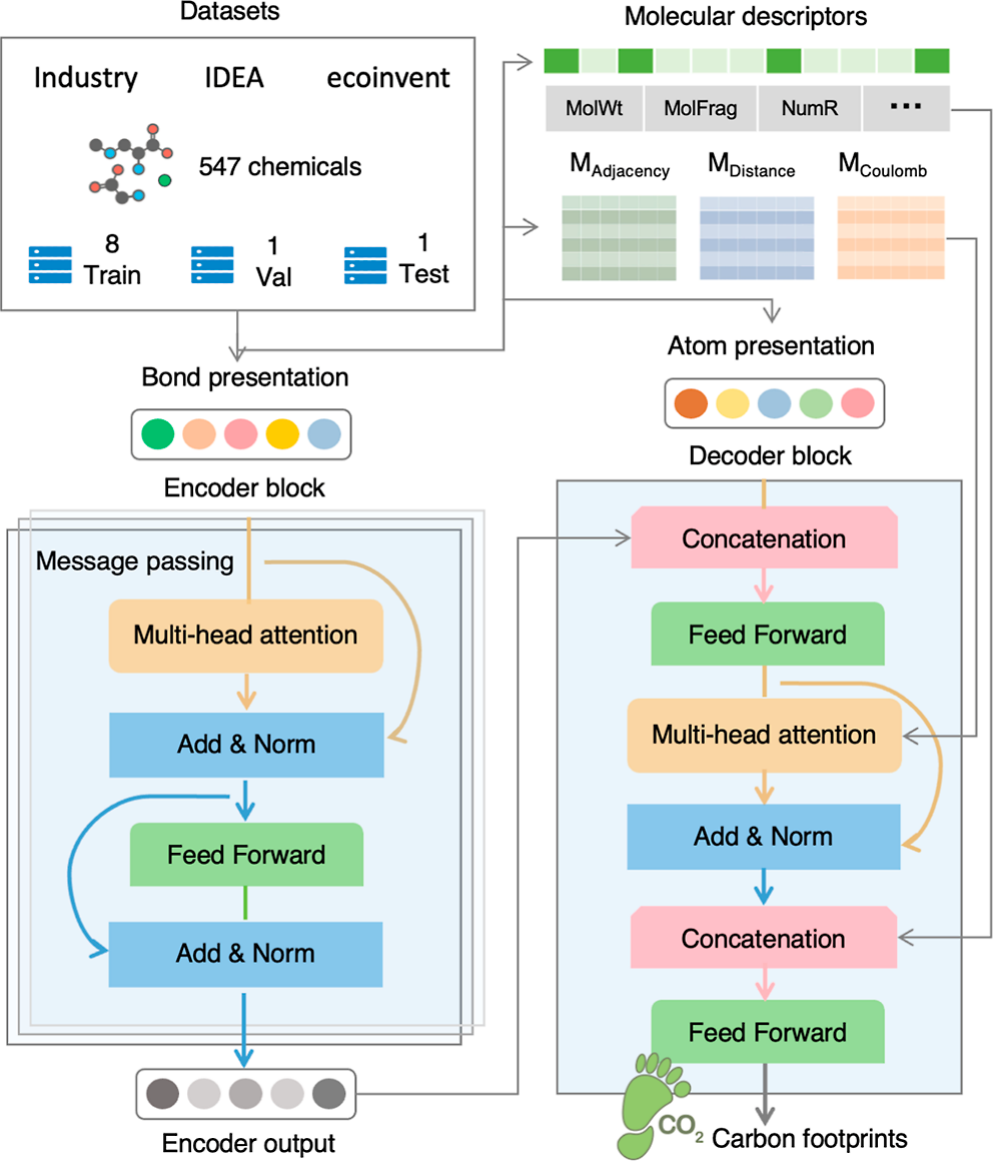
Development of FineChem 2 for estimating carbon footprints of chemicals. The data set is collected from the chemical industry, IDEA, and ecoinvent and is segregated into training, validation, and test data sets at 80, 10, and 10%, respectively. The bond and atom presentation of chemicals are extracted from the molecular structures, and the molecular descriptors, such as exact weights, types of molecular fragments, and number of rings, and the interatomic matrices, including adjacency, distance, and Coulomb matrixes, were also generated as the model’s input. The extracted bond feature matrix is first processed through a self-attention layer and bond update functions in the message-passing layer and then combined with the atom feature matrix. Next, the combined matrix is input into a self-attention layer in conjunction with interatomic matrices to extract hidden interactions between the bonds and the atoms. Finally, the learned features are concatenated with the list of precalculated molecular descriptors and then entered into feed-forward layers to achieve the final output. Add: residual connection; Norm: layer normalization.

Molecular representations at the bond, atomic, and molecular levels were extracted via graph neural networks.^33^ The molecular structures of chemicals were considered as graphs *G* = (ν,ϵ). We used one-hot vectors to represent categorical features of atoms and bonds, which is a process of converting numerical variables to categorical data variables and can make categorical data more expressive and ensure that ML models do not assume that higher numbers are more important.^34^ Each atom *v*_*i*_∈ν was represented by a one-hot vector, representing its atom types, degrees, chirality, hybridization types, number of hydrogen atoms attached, and aromaticity. Each bond (*v*_*i*_,*v*_*j*_) ∈ ϵ was represented by a one-hot vector representing the bond types, stereochemistry properties, and surrounding substructures. In addition, to enhance the model’s predictive ability on small-scale data sets, we calculated ∼200 molecular descriptors, including exact weights and types of molecular fragments, using the RDKit and DescriptaStorus, a Python package designed for generating molecular descriptors, and used those descriptors as model’s input. Interatomic matrices, including adjacency, distance, and Coulomb matrices, were also generated as the model inputs because they can potentially represent functional groups that have been proven relevant to the PCFs of chemicals (Figure 1).^13^

The atom-bond transformer framework was adopted to develop the FineChem 2 model (Figure 1).^35^ Compared with conventional algorithms, it has a unique feature that combines message-passing neural networks with a self-attention mechanism, which has been proven to have a better representation ability of molecules.^35^ To enhance the representation of molecules, bond features were extracted via message-passing layers and then updated in the self-attention layers. In message-passing networks, each bond was initialized with feature vectors, and each bond feature was updated by summing neighboring hidden states from the previous iteration.^36^ Next, bond message was processed by the multihead self-attention mechanism.^37^ The multihead self-attention block consisted of six heads, where each head was composed of two layers. The first layer implemented the self-attention mechanism,^37^ and the second layer was a fully connected feed-forward network with rectified linear unit activation.^38^ Then, the atomic features were obtained by summing the bond features, followed by the concatenation of the atom feature matrix and a self-attention layer in the decoder that also consisted of six identical heads. Subsequently, the adjacency, distance, and Coulomb matrices were incorporated into the model to provide electrostatic and structural characteristics of chemicals. Finally, the learned features were aggregated and concatenated with precalculated molecular descriptors for PCF estimation. The self-attention weights of all decoder blocks were summarized and assigned to the atoms to present the hidden knowledge that the model had learned to elucidate important substructures.

Adaptive moment estimation was used for optimization, which is an extension of stochastic gradient descent that is based on adaptive estimation of the first and second moments.^39^ The models adopted the adaptive moment estimation optimizer because it converges faster than conventional optimizers. To improve the model performance, its hyperparameters were optimized using the Bayesian optimization.^40^ Four hyperparameters of the model were optimized: message-passing iteration [1, 10] (interval: 1), interatomic feature scaler [0, 0.5] (interval: 0.05), dropout probability [0, 0.5] (interval: 0.05), and hidden dimension [100, 3000] (interval: 50). The model was constructed by using PyTorch 1.11.

### 2.3 ML Framework Evaluation

The data set was segregated into training, validation, and test data sets at 80, 10, and 10%, respectively, using two methods: random splitting and scaffold splitting. The validation set was used to find the optimal hyperparameters, while the test set was used to test the model’s performance. The segregation was repeated five times to demonstrate the robustness of the deep learning model. Root-mean-square error (RMSE) and mean and median absolute percentage errors (PEs) were employed to evaluate the performance of the ML models.
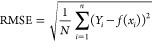
1
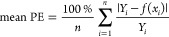
2
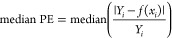
3

To evaluate the improvement of FineChem 2 in comparison with commonly used ML frameworks, nine baseline ML models were additionally developed using different combinations of three ML algorithms—ANN,^41^ random forest (RF),^42^ and support vector machine (SVM)^43^—and three types of molecular descriptors—the molecular access system (MACCS) fingerprint, RDKit fingerprint, and extended connectivity fingerprint with a diameter of 4 (ECFP4).^44^ Subsequently, 5-fold grid searches were conducted to determine the optimal hyperparameters. The baseline models were implemented using scikit-learn 1.2.1 and RDKit 2019.09.03 (see Supporting Information for details).

### 2.4 Benchmarking FineChem 2 with Previous Pre-LCA Tools

An external data set of 16 randomly selected chemicals from the chemical industry that were not included in any model development was used to deliver an unbiased and rigorous benchmark study. It included four chemicals with a relatively high PCF (>10 kg CO_2_-equiv/kg) and high complexity, seven chemicals with a moderate PCF (5–10 kg CO_2_-equiv/kg) and diverse structures, and five chemicals with a low PCF (<5 kg CO_2_-equiv/kg) that were mostly linear molecules with simple structures. Three representative pre-LCA tools that were developed based on features extracted from the molecular structures were reproduced, including FineChem 1,^13^ ANN-DP,^15^ and rapid-ANN.^16^ The predictive abilities of FineChem 2 and the three existing pre-LCA tools were evaluated using the aforementioned external data set (see Supporting Information for details).

### 2.5 Model Interpretability Evaluation

Three representative chemicals (one linear, one with a ring structure, and one complex) were selected as case studies to evaluate and demonstrate the applicability of FineChem 2 for identifying PCF-intensive substructures: butyl acrylate, 1-(2-hydroxyethyl)piperazine, and bis(2-ethylhexyl) terephthalate. To interpret which chemical substructures are mostly important to the prediction and contribute to their PCFs, the attention weights of all decoder blocks were summarized, assigned to atoms, and visualized using RDKit. The synthesis reactions and corresponding raw materials of these three chemicals were retrieved from the Reaxys database. The attention weights were also used to infer the critical raw materials for production that contribute the most to their PCFs. The predicted relative contribution of individual raw materials to the PCF of the final chemical was compared with their PCFs calculated based on standard LCA to evaluate the accuracy of the predicted results.

### 2.6 Evaluation of the Applicability Domain of Pre-LCA Tools

To evaluate the chemical space where FineChem 2 can make reliable predictions, a comprehensive list of chemicals was obtained from chemical databases. High-production-volume (HPV) chemicals were collected from the database of existing chemicals of the Organization for Economic Co-operation and Development (OECD). Daily chemicals (chemicals in daily use products, e.g., cosmetics, shampoo, toothpaste, body wash, and dish soap) and food additives were obtained from MolBase, a comprehensive chemical e-commerce platform. The plastic additives and processing aids were obtained from a previous study.^11^ The molecular structures of the chemicals were retrieved from PubChem^27^ and ChEMBL.^28^ Redundant data, e.g., the same organic chemicals with different ions, were merged according to normalized SMILES, and metal ions in chemicals were removed using RDKit. Furthermore, mixtures, polymers, and inorganic chemicals were removed.

A previously reported approach based on the Euclidean distance and *k*-nearest neighbors was used to quantify the AD of pre-LCA tools.^45^ First, the threshold *T* for determining whether a chemical was within the AD was calculated on the training set according to the previous approach, which is defined as
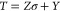
4where σ is the standard deviation, *Y* is the average of the Euclidean distances of chemicals in the training set, and *Z* is an empirical parameter to control the significance level. *Z* was set to 0.5 according to the suggestion of the previous study.^45^ Subsequently, the average Euclidean distance between the query chemical and *k*-most similar chemicals in the training data set was calculated. *k* = 5 was selected according to the suggestion of OECD.^46^

The Euclidean distance is defined as
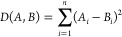
5where *D* is the distance between chemicals A and B, and *A*_*i*_ and *B*_*i*_ are the *i*_th_ molecular descriptors. The ECFP fingerprint was used as the molecular descriptor owing to its simplicity.

If the distance was above the threshold, the query chemical was considered outside the AD; otherwise, it was considered within the AD. The previous pre-LCA tools were mostly developed based on chemicals in ecoinvent or a part of ecoinvent.^15−17^ Therefore, organic chemicals in ecoinvent used in a previous study^15^ were collected to calculate the AD of the previous pre-LCA tools for comparison.

## 3 Results and Discussion

### 3.1 Construction of a Comprehensive Data Set for Predicting PCFs

1108 data sets of organic chemicals were obtained from ecoinvent (Table S1), IDEA (Table S2), and the industry. After data cleaning, they accounted for the PCFs of 547 unique organic chemicals, which are significantly larger than the data sets used in previous studies (Figure S1).^13,15−17,47^ Most data from the industry have not been included in any public LCA databases and have resulted in a structurally more diverse training data set than previous works (Figure S2). In addition, chemicals in our new data set have more widely distributed physicochemical proprieties and PCFs than the chemicals that were previously used for modeling (Figures S1 and S3).^15^ The percentage of chemicals with PCF >8 kg CO_2_-equiv/kg in the new data set is 20.3%, whereas that in the ecoinvent is 9.6%. 63 unique molecular scaffolds are identified in our data set, which shows a ∼3-fold improvement over those used in previous studies. The improvement in the training data set provided an opportunity to use more delicate ML algorithms in FineChem 2 and a basis for predicting the PCFs of fine chemicals with more complex structures.

### 3.2 Benchmarking FineChem 2 with the Baseline ML Models

**Figure 2:**
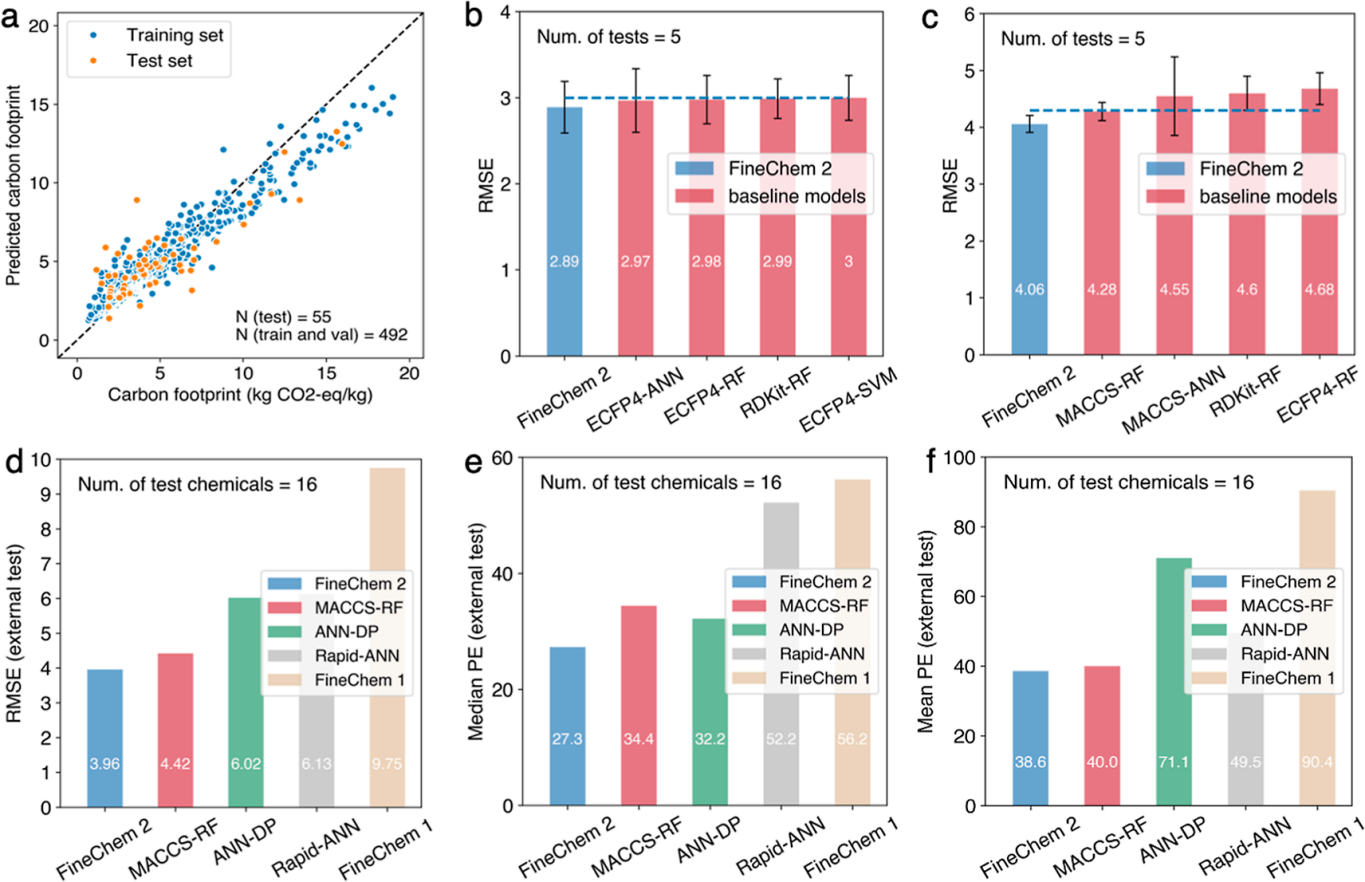
Evaluation of model performances for carbon footprint prediction. (a) Performance of the FineChem 2 on training and test sets. (b) RMSE of FineChem 2 and top four baseline ML models when using random splitting. (c) RMSE of FineChem 2 and top four baseline ML models when using scaffold splitting. RMSE (d), median PEs (e), and mean PEs (f) of pre-LCA tools on the external test set.

Good predictive accuracy of FineChem 2 is observed for both training and test data sets (Figure 2a). For the randomly segregated test sets, it obtains an RMSE of 2.89 kg CO_2_-equiv/kg. In comparison with the nine baseline models based on the commonly used ML algorithms and molecular descriptors, FineChem 2 exhibits the best performance (RMSE = 2.89), followed by ECFP4-ANN (RMSE = 2.97), ECFP4-RF (RMSE = 2.98), and RDKit-RF (RMSE = 2.99; Table S3 and Figure 2b).

A major limitation of previous pre-LCA tools is their weak predictive ability for chemicals with structures that differ from those of the training set because of the relatively weak extrapolation ability of the algorithms used. To evaluate the predictive ability, the performance of the ML models was tested by using chemicals with new scaffolds. As a result, despite the performances of all models being compromised, FineChem 2 still achieves the best performance (RMSE = 4.06), illustrating that it has better extrapolation ability than the other conventional ML algorithms (Table S3 and Figure 2c).

### 3.3 Benchmarking FineChem 2 with the Previous Pre-LCA Tools

When compared with the previous tools, FineChem 2 featured the lowest values for various evaluation metrics, including median PE (27.3%), mean PE (38.6%), and RMSE (3.96), thanks to the high-quality training data and the state-of-the-art ML algorithm (Figure 2d–f). FineChem 2 displays an improvement of the RMSE, median PE, and mean PE by ∼55% compared to FineChem 1. When compared to the recently released rapid-ANN^16^ and ANN-DP,^15^ the performance of FineChem 2 generally improved by ∼30%.

The predictive ability of FineChem 2 and the previous pre-LCA tools is presented in Figures S4 and S5. All tools struggled when predicting chemicals with relatively high or extremely low PCFs because the information extracted from the molecular structures may not fully represent the complex chemical production processes. For simple chemicals (e.g., ethylene) with extremely low PCFs, different feedstock types (e.g., petroleum, natural gas, and coal) with different emission profiles are a major determinant of the impact and, hence, cause large variability in the data. As these chemicals are usually covered by available data, pre-LCA tools should not be used for them. For chemicals with high PCFs, multiple synthesis routes may exist, and extensive purification may sometimes be required, thus increasing the variability of the PCFs. At the same time, the availability of training data sets for complex chemicals with molecular weight >800 Da is still low, which may limit the predictive ability of ML tools for more complex substances. For chemicals with moderate PCFs (5–10 kg CO_2_-equiv/kg), FineChem 2 demonstrates a more robust performance, whereas the performance of the other tools is perturbed drastically. ANN-DP^15^ achieves relatively lower median PE than the other previous pre-LCA tools mostly because it used a special data processing strategy: developing a specific ML model for each individual test chemical based only on 60% of the most similar chemicals in the entire training set.^15^ The median PE of ANN-DP is 32.2%, while the mean PE is 71.1%, indicating that the data processing strategy is useful for most of the chemicals tested. But from another perspective, such a data processing strategy limits the extrapolation ability of the model, which restricts the model AD to basic chemicals similar to those with known PCFs. The rapid-ANN^16^ was developed based on the standard ML procedure and chemical data from ecoinvent. It presents the overall stable errors for chemicals with different PCFs and complexities. However, the median and mean PEs of the external test set are both approximately 50%, in contrast to those of FineChem 2 being 27.3 and 38.6%, respectively (Figure 2e,f).

The evaluations of the external data set demonstrate that FineChem 2 is more accurate and reliable than the previous pre-LCA tools for predicting the PCFs of chemicals. To further elucidate reasons for the improvements of FineChem 2, the performance of the baseline model with the best extrapolation ability, MACCS-RF, was tested using the same external data set. Despite being trained on the same data set, the baseline model demonstrates higher RMSE and median PE when compared with FineChem 2 (Figures 2d,e and S4), indicating that changes on both algorithm and data set levels are needed to make a better prediction.

### 3.4 Identification of the PCF-Intensive Substructures and Critical Raw Materials

ML models are usually considered “black boxes” due to their poor interpretability.^23,48^ In response, previous studies have used a SHAP-based approach^24^ to identify the most important molecular descriptors that affect the PCFs of chemicals.^15^ However, it could not provide clear insights on, for example, PCF-intensive substructures and raw materials to guide the design of more sustainable molecules and processes. The attention mechanism has been used to identify important features that affect the activity of molecules, enzymes, and reactions.^22,49^ FineChem 2 employs the attention mechanism to capture the contributions of substructures in a chemical to its PCF, thereby promoting learned knowledge and improving the model interpretability.

**Figure 3:**
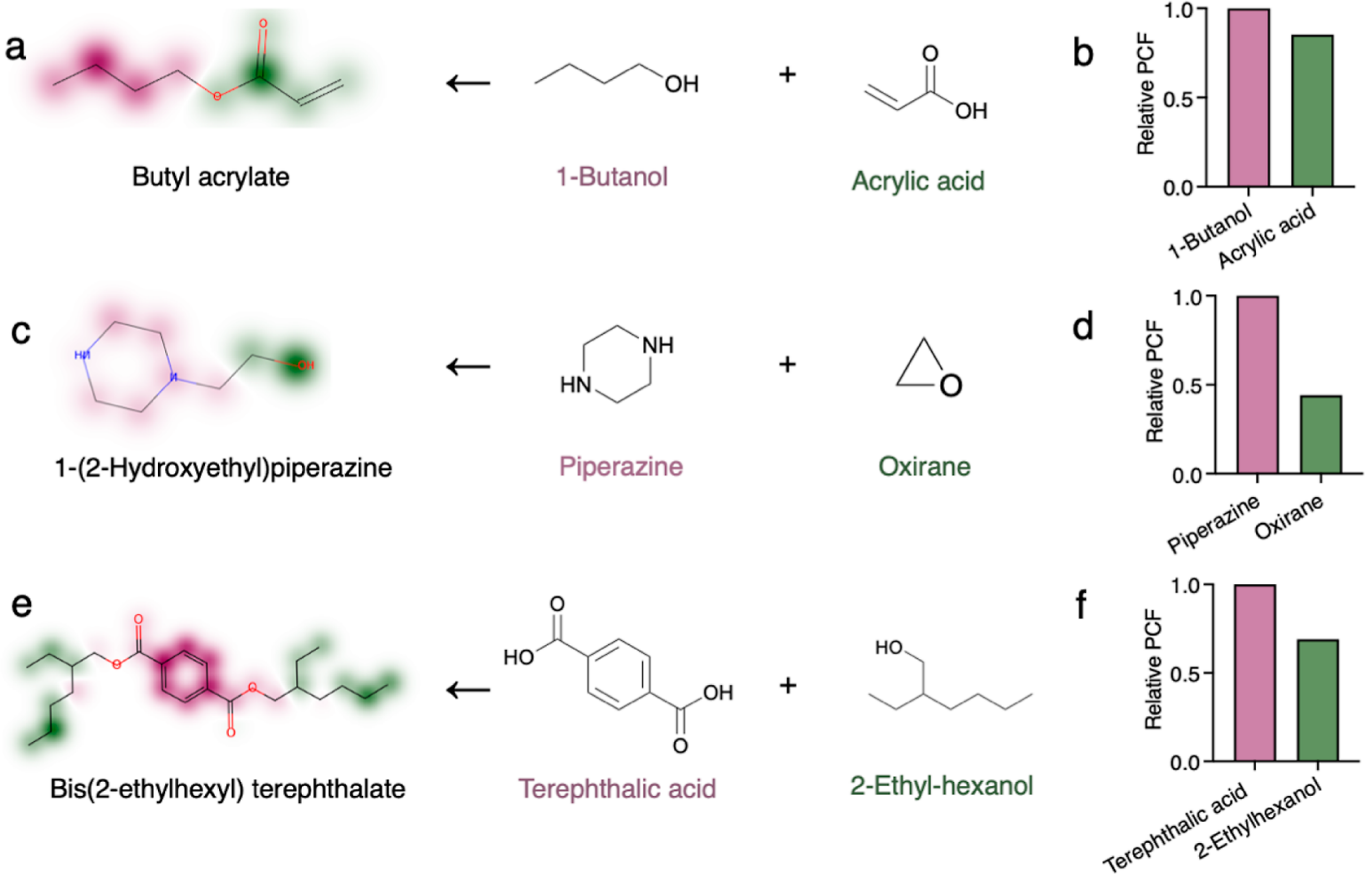
Identification of PCF-intensive substructures and critical raw materials with the attention mechanism of FineChem 2. (a,c,e) Chemicals’ attention weights and synthesis reactions. Red areas show the corresponding substructures that have a relatively higher PCF contribution, while green areas show the substructures that have a relatively lower contribution. (b,d,f) Relative PCF of raw materials of the tested synthesis reactions calculated by standard LCA and data from the chemical industry and IDEA v2.3.

To demonstrate this ability of FineChem 2 in identifying the PCF-intensive substructures, the attention weights of butyl acrylate, 1-(2-hydroxyethyl)piperazine, and bis(2-ethylhexyl) terephthalate were mapped to the corresponding atoms, and the relative PCF contribution of each substructure was identified (Figure 3). Butyl acrylate is a linear molecule that is commonly synthesized by esterification of 1-butanol and acrylic acid.^50^ The red contour of the alcohol indicates that the PCF of butyl acrylate originated primarily from 1-butanol (Figure 3a). Using standard LCA, we found that the PCF of butanol was higher than that of acrylic acid (Figure 3b), which was consistent with the inference of FineChem 2.

For more complex molecules, such as those with rings and side chains, FineChem 2 may also achieve meaningful results. 1-(2-Hydroxyethyl)piperazine is a HPV intermediate used in the manufacturing of surfactants, synthetic fibers, and pharmaceuticals. It is usually synthesized from piperazine and oxirane (Figure 3c).^51^ Bis(2-ethylhexyl) terephthalate is a diester of terephthalic acid and branched-chain 2-ethyl-hexanol (Figure 3e).^52^ FineChem 2 successfully predicts that their PCFs were primarily derived from piperazine (Figure 3c,d) and terephthalic acid (Figure 3e,f), respectively.

Compared to the SHAP-based approach, FineChem 2 can provide more direct insights at the atomic level to guide the design of more sustainable molecules. In addition, it could also assist in tracking the relative PCF contribution along supply chains when detailed process data are unavailable, thereby identifying critical raw materials and intermediates for future improvements. With these insights, strategies such as structural modification of PCF-intensive substructures or replacing the corresponding raw materials with more sustainable alternatives can be specifically applied.^53^

### 3.5 Validation of the Applicability Domain of FineChem 2

**Figure 4:**
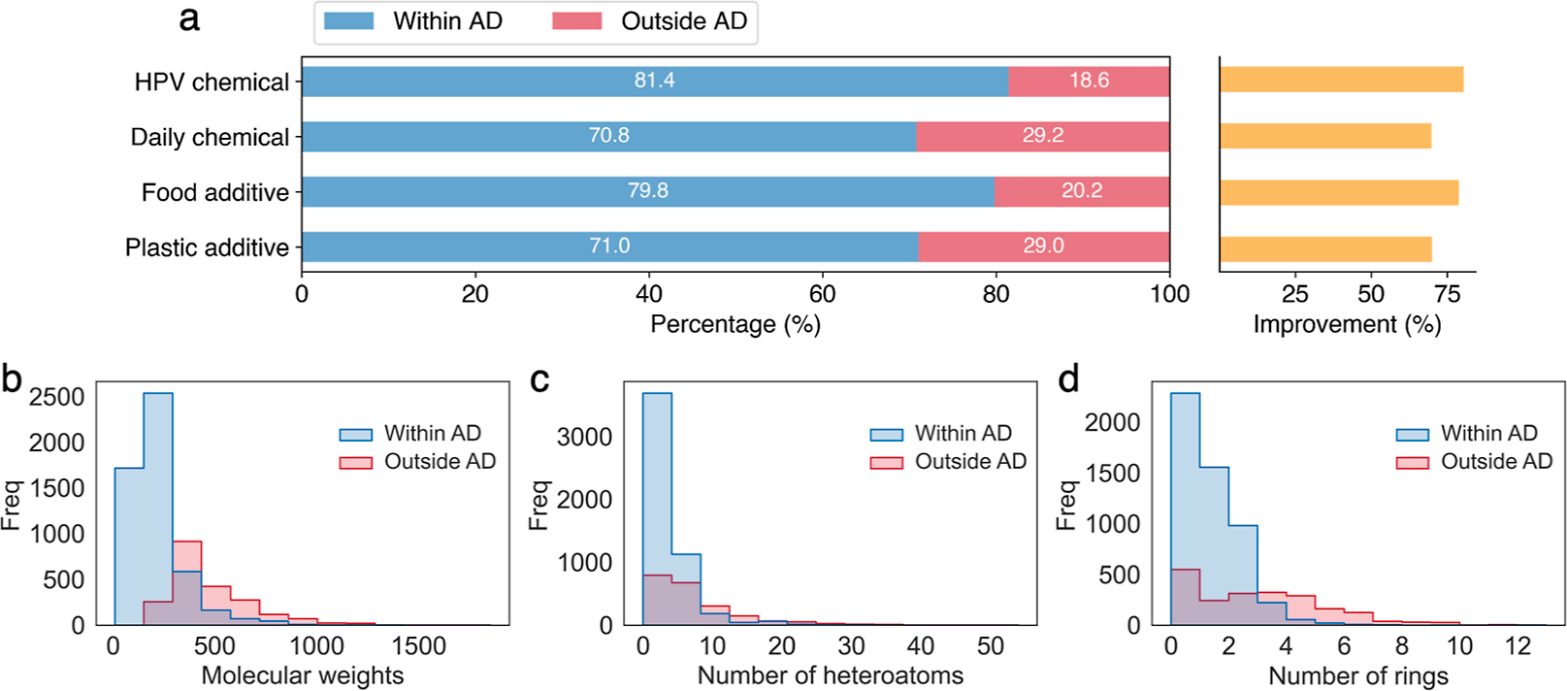
AD of FineChem 2 on HPV chemicals (*N* = 2502), chemicals in daily use products (*N* = 1589), food additives (*N* = 506), and plastic additives (*N* = 5281). The blue area indicates the percentage of chemicals within AD, while the red area indicates outside AD. Orange bars indicate the relative improvement of AD in comparison with previous pre-LCA tools developed based on ecoinvent data. The distribution of molecular weights (b), number of heteroatoms (c), and number of rings (d) of all collected chemicals (*N* = 7254) within and outside the AD of FineChem 2.

Based on the Euclidean distance-based approach, FineChem 2 is applicable to 81.4% of the 2502 organic HPV chemicals listed by the OECD, which significantly expands by eight times compared to existing LCA databases (Figures 4a and S6). This is critical, as only ∼10% of the organic HPV chemicals have been included in the ecoinvent v3.8 and IDEA v2.3 databases, demonstrating major data gaps for chemicals in LCA (Figure S4).

Chemicals are present in more than 90% of manufactured goods.^1^ One major data gap in the PCF calculations, for example, for plastics, food products, and daily necessities, is the lack of data on chemical additives and processing aids. Because of data unavailability, the chemical additives and processing aids therein are usually neglected or only crudely estimated in sustainability studies, while they may contribute significantly to the PCFs. In our evaluation, 79.8% of the 506 food additives, 71.0% of the 5281 plastic additives, and 70.8% of the 1589 daily chemicals are within the AD of FineChem 2 (Figure 4a), indicating that FineChem 2 can serve as an effective tool to fill the data gaps in LCA. Thanks to the diverse and more comprehensive training data, the AD of FineChem 2 for all tested organic chemicals (*N* = 7254 after removing repetitive chemicals) has improved overall by ∼75%, compared with the previous tools (Figures 4a and S7). However, most chemicals with molecular weights greater than 500, number of heteroatoms over 10, or number of rings over 4 still lie outside the AD of FineChem 2 (Figures 4b,d and S8), requiring further expansion of the training sets.

## 4 Limitations and Outlook

With increasing efforts and attention toward designing sustainable molecules, estimations are indispensable to fill in the data gaps of chemical sustainability assessment. In this study, we construct a comprehensive and high-quality training data set and thus develop FineChem 2 for estimating the PCFs of diverse chemicals. FineChem 2 performs well on the randomly segregated test sets and exhibits significantly better robustness than the baseline models for chemicals with new scaffolds. Among the pre-LCA tools, FineChem 2 shows the best performance in terms of various evaluation metrics. FineChem 2 also exhibits a good interpretability. The attention mechanism enables FineChem 2 to successfully identify the PCF-intensive substructures and raw materials, which may aid in the design of more sustainable molecules and synthesis routes, including selection of raw materials.

Several aspects of this study could be expanded further. First, current molecular structure-based models, including FineChem 2, are applicable only to pure organic chemicals. For polymers, mixtures, and inorganic chemicals, different algorithms and molecular descriptors are required; therefore, these are not included in this study. Second, the production of specialty chemicals such as pharmaceuticals requires extensive purification processes; therefore, they usually have remarkably high PCFs. The PCF contribution of the purification process could not be fully represented in the molecular structures. Therefore, molecular structure-based models are not ideal for predicting their PCFs. Finally, chemicals synthesized via different routes and produced in different regions may have different PCFs. Although molecular structure-based models have the lowest data requirements and the highest simplicity, the variability caused by different synthesis routes and production processes cannot be reflected in the molecular structures. In the future, new pre-LCA models or modeling strategies will be required to take into account different synthesis routes and production processes to achieve more accurate predictions.

Nevertheless, we are confident that the current study provides an effective approach for estimating the PCFs of chemicals at early design stages, which can considerably bridge the data gaps in LCA^21^ and assist in sustainable molecules design^54^ and chemical engineering.^55^ Moreover, the general framework used by FineChem 2 can be readily adopted into prediction tasks other than PCFs, such as predictions of energy demands and environmental toxicity, when high-quality training data are available.

## Data Availability

Chemical production data was collected from ecoinvent v3.8 (https://ecoinvent.org/), IDEA v2.3 (https://idea-lca.com/en/), and anonymous industrial partners. User licenses are required to access the ecoinvent and IDEA databases. Data from industrial partners is strictly confidential upon the nondisclosure agreement. Chemicals used for AD analysis were collected from MolBase (https://www.molbase.cn/), OECD’s existing chemical database (https://hpvchemicals.oecd.org/), and a previous study (https://pubs.acs.org/doi/10.1021/acs.est.1c00976). Code for producing all figures is available from a Zenodo repository (10.5281/zenodo.10410702). Additional information required to reanalyze the data reported in this paper is available from the corresponding author upon reasonable request.

## Supporting Information Available

Detailed results of the development and evaluation of the pre-LCA models (PDF)
