# Supplementary material for: Enhanced Deep-Learning Model for Carbon Footprints of Chemicals
Source: ACS Sustain Chem Eng. 2024 Feb 5;12(7):2700–8. doi: 10.1021/acssuschemeng.3c07038 (PMC10880087; doi:10.1021/acssuschemeng.3c07038)
Supplement: Supplementary file 1 — sc3c07038_si_001.pdf [file sc3c07038_si_001.pdf]

Supporting information

Enhanced deep-learning model for carbon footprints of chemicals

Dachuan Zhang,*[a] Zhanyun Wang,[ab] Christopher Oberschelp,[a] Eric Bradford,[a] and Stefanie Hellweg[a]

[a] National Centre of Competence in Research (NCCR) Catalysis, Ecological Systems Design, Institute of Environmental Engineering, ETH Zürich, 8093 Zürich, Switzerland

[b] Technology and Society Laboratory, Empa-Swiss Federal Laboratories for Materials Science and Technology, St. Gallen CH-9014, Switzerland

*Corresponding author:

Dachuan Zhang, dachuan.zhang@ifu.baug.ethz.ch

Number of pages: 13

Number of figures: 8

Number of tables: 3

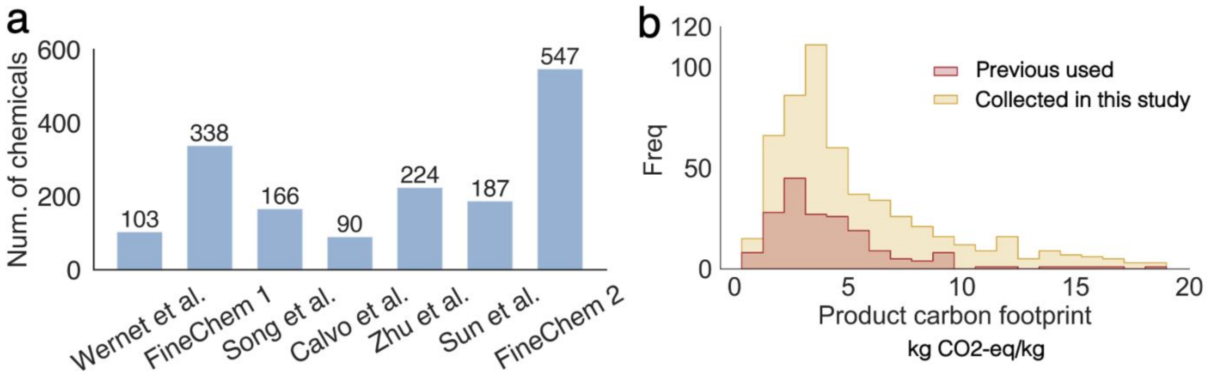

Figure S1. Comparison of datasets for pre-LCA model development. (a) Number of chemicals used for modeling in this work and previous studies. (b) The PCF distribution of chemicals previously used for modeling and the new dataset used for developing FineChem 2.

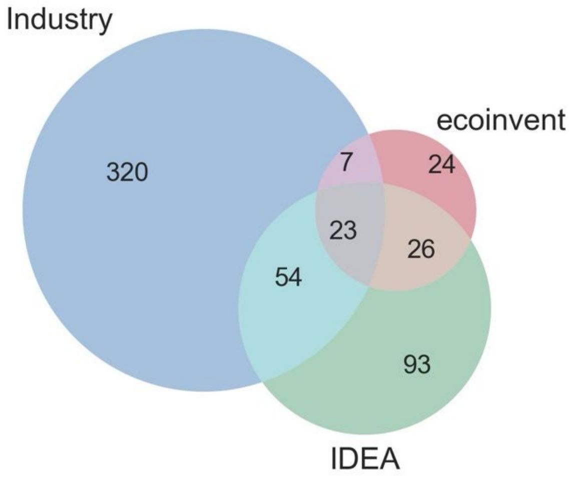

Figure S2. The Venn plot of organic chemicals collected from the chemical industry, IDEA v2.3, and ecoinvent v3.8. For ecoinvent, datasets contaminated with direct proxy data or with major proxy use in their educts were excluded. Only 80 datasets from ecoinvent with high quality were included for modeling.

Table S1. Selected high-quality datasets from ecoinvent for model development.

High-quality datasets from ecoinvent without direct proxy used				
Methanol	2-Butanol	Formic acid	Propanal	Dioxane
Adipic acid	Trichloropropane	1-Butanol	Cyclohexane	4-Methyl-2-pentanone
Hydrogen cyanide	Isobutanol	Methyl methacrylate	Maleic anhydride	Toluene diisocyanate
Diethyl ether	Tetrahydrofuran	Dichloromethane	Acrylonitrile	Acetylene
Glycerine	Methyl formate	Methyl ethyl ketone	Butane	Isobutyl acetate
Formaldehyde	P-nitrotoluene	Ethyl acetate	Trichloromethane	Benzene
Isohexane	Methylamine	Acetonitrile	Phthalic anhydride	Acetone cyanohydrin
Vinyl fluoride	Benzyl alcohol	Tetrachloroethylene	Propane	Chloronitrobenzene
Methylcyclohexane	Vinyl chloride	Ethane	Acetic anhydride	Butane-1,4-diol
Butyl acrylate	Benzal chloride	Isobutane	Ethanol	Monochlorobenzene
Ethylene	Methylchloride	Toluene	2-Methyl-2-butanol	Propyl amine
1-Propanol	Methyl acetate	Propylene	Pentane	Oxalic acid
Urea	Isopropyl acetate	Acetic acid	2-Cyclopentone	Dimethyl malonate
Epichlorohydrin	Ethylene carbonate	Allyl chloride	Chloroacetic acid	1,1-Difluoroethane
Ethyl tert-butyl ether	P-dichlorobenzene	Benzaldehyde	Toluene diisocyanate	Cyanoacetic acid
Acetaldehyde	O-dichlorobenzene	Benzyl chloride	Acetone	2-Nitrophenol

Table S2. Selected datasets from IDEA for model development.

IDEA datasets used for model development				
Methanol	2-butanol	Formic acid	Adipic acid	1,2,3-Trichloropropane
1-Butanol	Cyclohexane	Hydrogen cyanide	Isobutanol	Methyl methacrylate
Maleic anhydride	Tetrahydrofuran	Methylene chloride	Acrylonitrile	Glycerol
Methyl formate	Methyl ethyl ketone	Formaldehyde	Ethyl acetate	Benzene
Monomethylamine	Phthalic anhydride	Acetone cyanohydrin	Tetrachloroethylene	Propane
Vinyl chloride	Acetic anhydride	1,4-Butanediol	n-Butyl acrylate	Isobutane
Ethanol	Monochlorobenzene	Ethylene	Methyl chloride	Propylene
Oxalic acid	Urea	Acetic acid	Epichlorohydrin	Allyl chloride
Monochloroacetic acid	Chlorine	Ethyl tert-butyl ether	p-Dichlorobenzene	Acetaldehyde
o-Dichlorobenzene	Acetone	Propionic acid	Monoethanolamine	Dimethylamine
Diethanolamine	Triethanolamine	Dimethylformamide	Morpholine	n-Methyl-2-pyrrolidone
Trimethylamine	Diethylenetriamine	Dibutylamine	Dipropylamine	Diethylene glycol monoethyl ether
Isopropyl alcohol	Methylene diphenyl diisocyanate	Nitrobenzene	Cyclohexanone	Aniline
2,4-Dinitrotoluene	Propylene oxide	Toluene-2,4-diamine	Cyclohexanol	Ethylene dichloride
Propylene glycol	Melamine	Tripropyleneglycol	Diethylene glycol	Hexamethylenediamine
Ethylbenzene	Ethylene oxide	n-Butyraldehyde	2-Ethylhexanol	1,3-Butadiene
Isobutylene	2-Ethylhexyl acrylate	n-Butyl acetate	Methyl acrylate	Isobutyraldehyde
2-Butoxyethanol	Diethylene glycol monobutyl ether	Dimethylacetamide	Triethylene glycol monobutyl ether	Acrolein
Ammonia	Acrylamide	gamma-Butyrolactone	2-Hydroxyethyl acrylate	o-Xylene
Hexamethylene diisocyanate	Methacrylic acid	Nitric acid	Ethylene glycol	Pentaerythritol
Citric acid	Isophthalic acid	Cumene	Pyridine	4-Methyl-1-pentene

Vinyl acetate	Caprolactam	Acrylic acid	1-Decene	Toluidine
3-Methylpyridine	Calcium carbide	Polypropylene	Phenol	Nonylphenol
1,1,1,3,3-Pentafluoropropane	Diphenyl carbonate	Propylene dichloride	Adiponitrile	1-Hexene
Triethylamine	Terephthalic acid	Dibutyl phthalate	Hydroquinone	Propylene tetramer
Malathion	1-Butene	Bisphenol A	m-Cresol	p-Cresol
Butyric acid	Triethylenetetramine	Carbon tetrachloride	Dimethyl ether	Methyl mercaptan
Allyl alcohol	1,1,1-Trichloroethane	Bis(2-ethylhexyl) phthalate	Trichlorofluorometh ane	Triethylene glycol monoethyl ether
DL-methionine	Tetrafluoroethylene	Pentaethylenehexami ne	Dichlorodifluoromet hane	Methyl isobutyl ketone
Tetraethylenepentam ine	1,4-Hexadiene	Valeric acid	Vinylidene chloride	Stearate
1,3-Dichloropropene	Bisphenol A diglycidyl ether	Ethyleneimine	Nonylphenol ethoxylate	Carbon disulfide
3,4-Dihydro-2H-1-benzopyran	Crotonic acid	Hydrobromic acid	Trichloroethylene	m-Xylene
Dimethyl terephthalate	Decabromodiphenyl oxide	Ethylene glycol monoethyl ether	Succinic acid	Methyl tert-butyl ether
Methane	Choline	2,6-Dimethylphenol	p-Methylstyrene	Paraldehyde
Trisphenol	Chlorodifluorometha ne	Dodecylbenzene	Fumaric acid	Sulfolane
ylphenyl phosphate	1-Octene	Dimethyl sulfoxide	Styrene	p-tert-Butylphenol
Sorbic acid	6-Hydroxyhexanoic acid	Glutaric acid	Phosgene	p-Xylene
Benzoic acid	1,1-Dichloro-1-fluoroethane	Dimethyl sulfide	Polyvinyl alcohol	Methyl salicylate
Salicylic acid	Toluene	Dibutyl oxalate	Hexamethylenetetra mine	Diethyl maleate

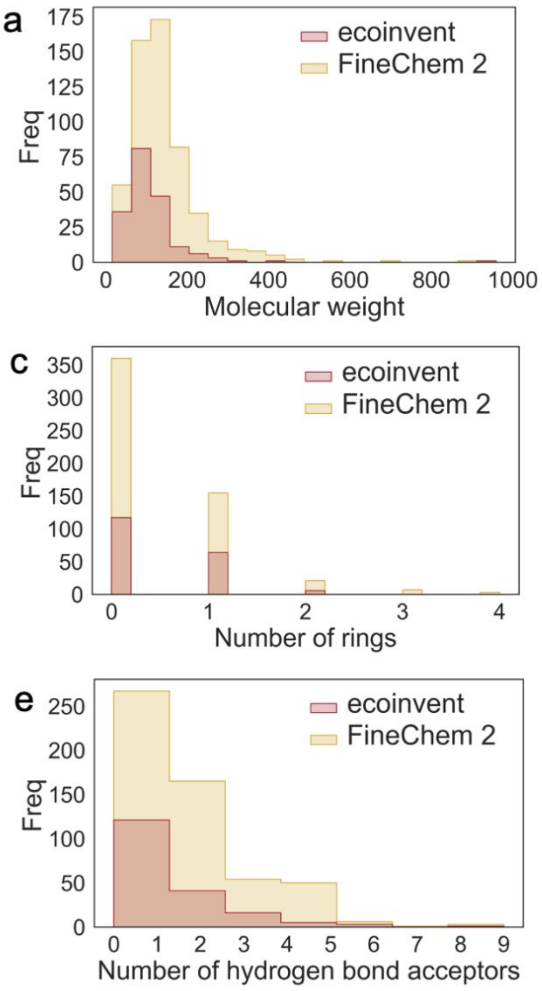

Figure S3. The distribution of physicochemical properties, including molecular weights (a), Slog*P* (b), number of rings (c), number of hydrogen bond donors (d), number of hydrogen bond acceptors (e), number of heteroatoms (f), of chemicals in ecoinvent that previously used for modeling and FineChem 2. FineChem 2 dataset has a more widespread distribution of physicochemical properties, which improves the applicability domain of FineChem 2 and enables FineChem 2 to have good predictive ability on structurally different chemicals.

S1. Comparison of the proposed model with baseline machine learning (ML) models

Nine ML models developed based on three common ML algorithms, including artificial neural network (ANN), random forest (RF), and support vector machine (SVM), and three types of molecular descriptors, including Molecular Access System (MACCS) fingerprint, RDKit fingerprint, and extended connectivity fingerprint with a diameter of 4 (ECFP4) were considered as baseline models. Two strategies were used to split test and training datasets, including random splitting and scaffold-based splitting. For random splitting, the training set and test set were generated randomly in a ratio of 9:1. Then, five-fold validation and grid search were conducted on the training set to find the optimal hyperparameters. For scaffold splitting, training, validation, and test datasets were generated in a ratio of 8:1:1 according to the scaffolds of chemicals. Then, grid searches were conducted on the validation set to find the optimal hyperparameters. Four hyper-parameters of the RF models were optimized, including the number of estimators [10, 1001] (interval: 200), min_samples_split [1, 101] (interval: 20), min_samples_leaf [1, 11] (interval: 2). Two hyper-parameters of the ANN models were optimized, including hidden layer sizes [100, (100, 30)], and solver types [adam, sgd, lbfgs]. Three hyper-parameters of the SVM models were optimized, including gamma [1e-3, 1e-4], penalty parameter [1, 10, 100, 1000], and kernel types [linear, rbf]. ML algorithms were implemented using scikit-learn 1.2.1, and molecular fingerprints were calculated by RDKit 2019.09.03.

Table S3. Root-mean-square errors of the proposed new model and baseline ML models

ML models	Random splitting	Scaffold-based splitting
FineChem 2 model	2.89±0.33	4.06±0.15
MACCS-SVM	3.51±0.41	5.19±0.59
MACCS-RF	3.51±0.41	4.28±0.16
MACCS-ANN	3.79±0.36	4.55±0.69
ECFP4-SVM	3.00±0.26	5.00±0.67
ECFP4-RF	2.98±0.28	4.68±0.28
ECFP4-ANN	2.97±0.37	4.74±0.37
RDKit-SVM	3.62±0.23	5.08±0.32
RDKit-RF	2.99±0.23	4.60±0.30
RDKit-ANN	3.57±0.25	5.08±0.60

S2. Benchmarking FineChem 2 with previous predictive life cycle assessment (pre-LCA) tools

An external dataset consisting of 16 chemicals from the chemical industry was randomly selected for benchmarking FineChem 2 with previous pre-LCA tools. The PCF of these chemicals ranges from ~1.5 kg CO_2-eq / kg to ~20 kg CO_2-eq / kg. Three representative pre-LCA tools were reproduced. Then, the performance of FineChem 2, MACCS-RF model (the baseline model with the best extrapolation ability), and previous pre-LCA tools were tested on the external dataset.

S3. Reproduction of FineChem 1

FineChem 1 was rewritten in Python according to our previous publication[1] and the R code provided online (https://emeritus.setg.ethz.ch/research/downloads/software---tools/fine-chem.html).

S4. Reproduction of the improved ANN models by data processing (ANN-DP)

The training dataset consisting of 189 chemicals and their PCFs was collected from the original publication[2]. For each chemical in the external dataset, a Euclidean distance-based approach was applied to screen the sub-dataset containing 60% of the most similar chemicals from the original training set for model development[2]. Padel-Descriptor[3] software was used to generate the molecular descriptors. Descriptors with a variance of zero were removed. One of any two features with high pairwise correlation (Pearson correlation coefficient >0.95) was removed. Then, features used for modeling were extracted by principal component analysis (PCA) that preserved 95% of the variance in the dataset. The learning rate was set to 0.001, the number of hidden layers was set to 2, the number of hidden neurons was set to 64, the activation function was set to ReLU, the optimizer function was set to Adam, batch size was set to 128 according to the original publication.

S5. Reproduction of Rapid ANN model for screening of LCA impacts (Rapid-ANN)

A dataset consisting of 166 chemicals was collected from the original publication[4]. The PCFs of chemicals were calculated according to the original publication. We used an open-access software, Padel-Descriptor[3] to calculate molecular descriptors because the software (a previous version of Dragon software) used to calculate molecular descriptors in the original publication is no longer available. Using open-access software also makes the results easier to be reproduced by other researchers. Then, features used for modeling were extracted by PCA that preserved 95% of the variance in the dataset. The learning rate was set to 0.001, the number of hidden layers was set to 2, the number of hidden neurons was set to 16, the activation function was set to ReLU, the optimizer function was set to Adam, the learning rate was set to 0.001, the learning approach was set to 500, and the regularization factor was set to 0.01 according to the original publication.

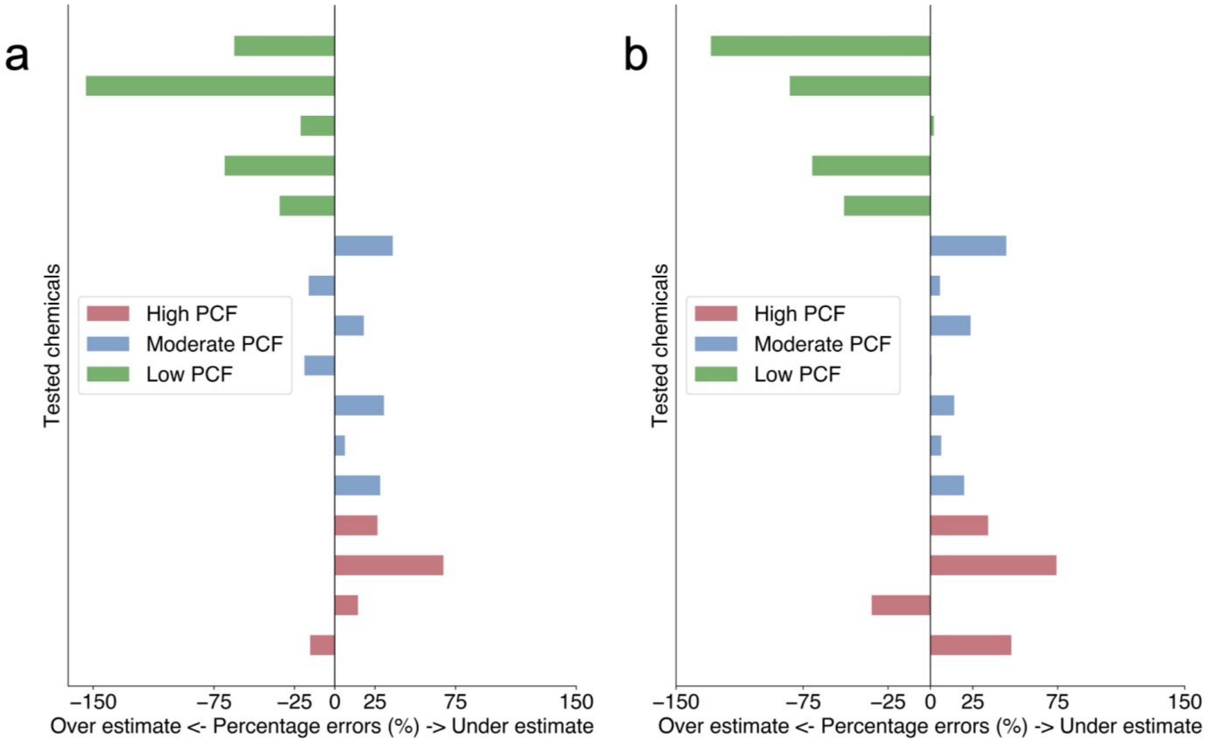

Figure S4. The performance of (a) FineChem 2 and (b) the MACCS-RF model on the external dataset (N=16). The red bars indicate chemicals with high PCF (PCF>10 kg CO2-eq/kg), blue indicates moderate PCF (10 kg CO2-eq/kg >PCF>5 kg CO2-eq/kg), and green indicates low PCF (PCF<5 kg CO2-eq/kg). A negative percentage error means the PCF was over-estimated, while a positive percentage error means the PCF was under-estimated.

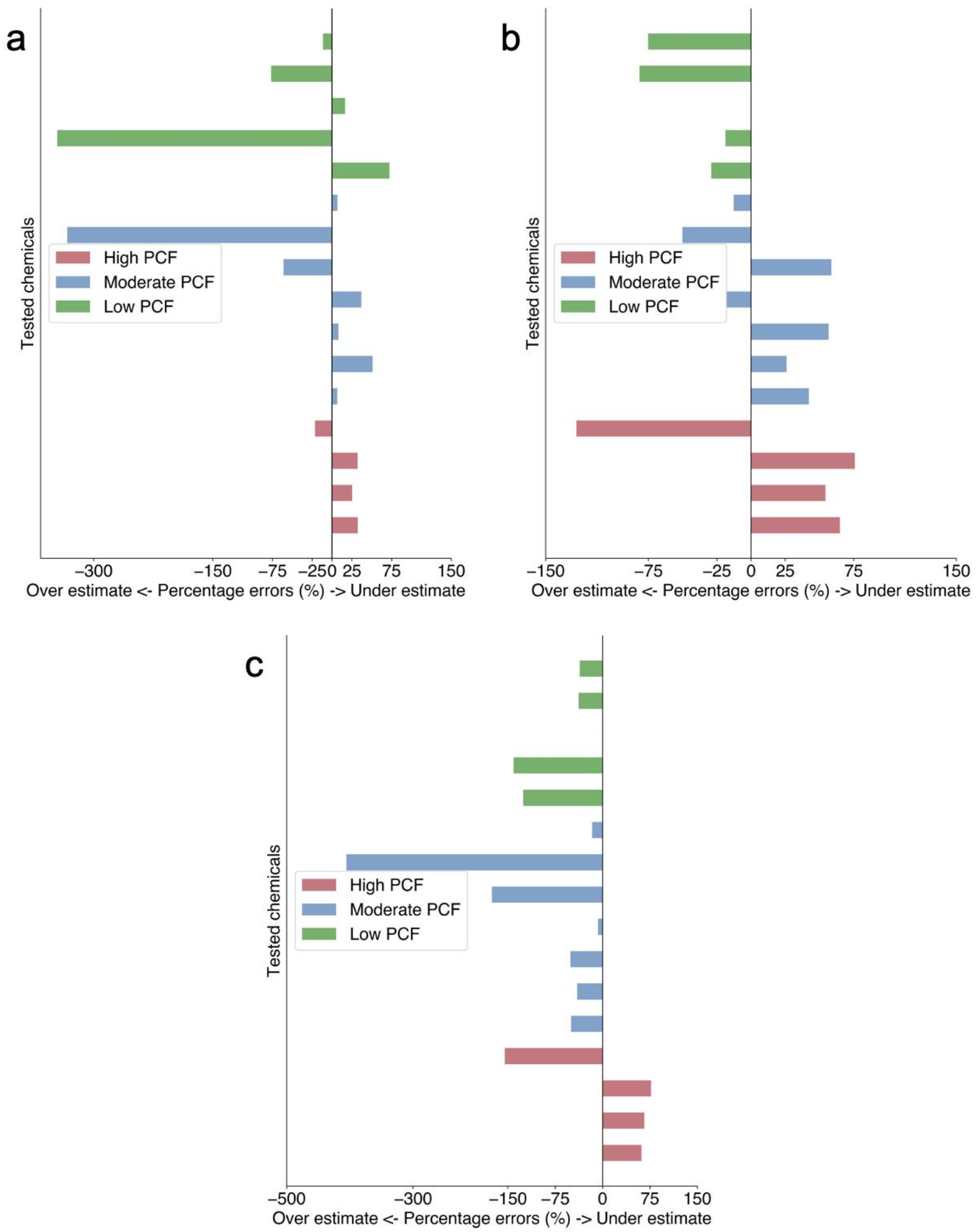

Figure S5. The performance of previous pre-LCA tools, including ANN-DP (a), Rapid-ANN (b), and FineChem 1 (c) on the external dataset (N=16). A negative percentage error means the PCF was over-estimated, while a positive percentage error means the PCF was under-estimated.

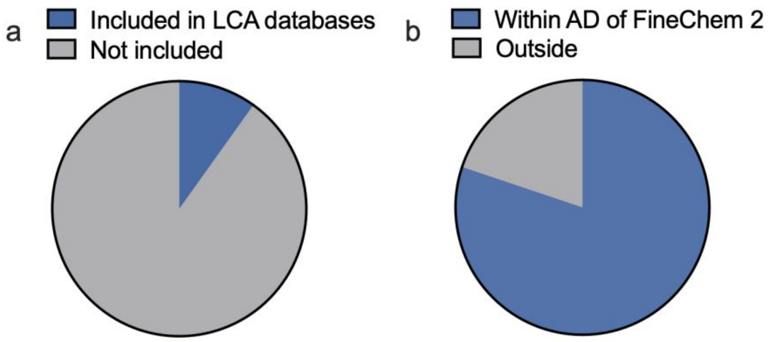

Figure S6. The coverage of organic high-production volume chemicals (N=2502) of LCA databases (ecoinvent v3.8 and IDEA v2.3) and the applicability domain (AD) of FineChem 2. (a) 9.9% of HPV chemicals have been included in ecoinvent and IDEA. (b) 81.4% of HPV chemicals are within the AD of FineChem 2.

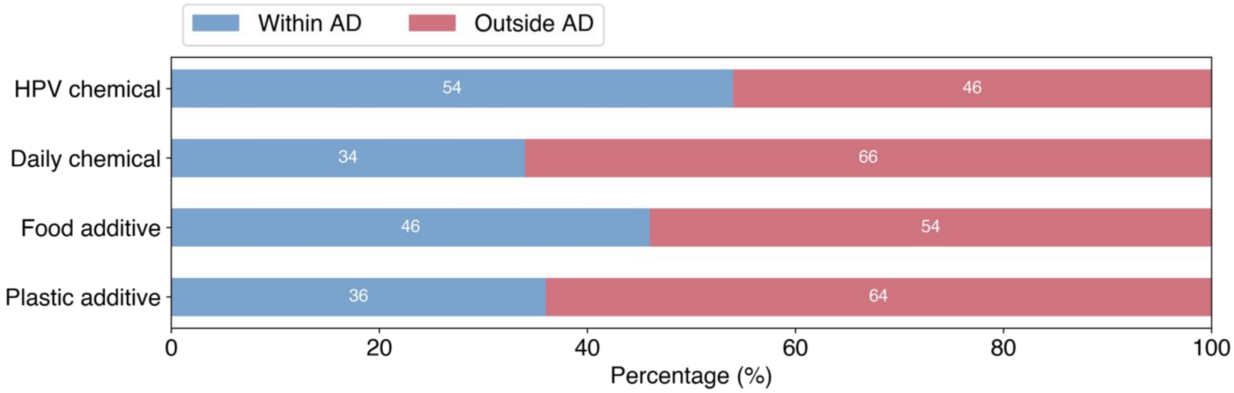

Figure S7. The applicability domains of ML models built on ecoinvent on high-production volume (HPV) chemicals (N=2502), daily chemicals (N=1589), plastic additives (N=5281), and food additives (N=506). The blue areas indicate the percentage of chemicals within the applicability domain, while the red areas indicate the percentage outside.

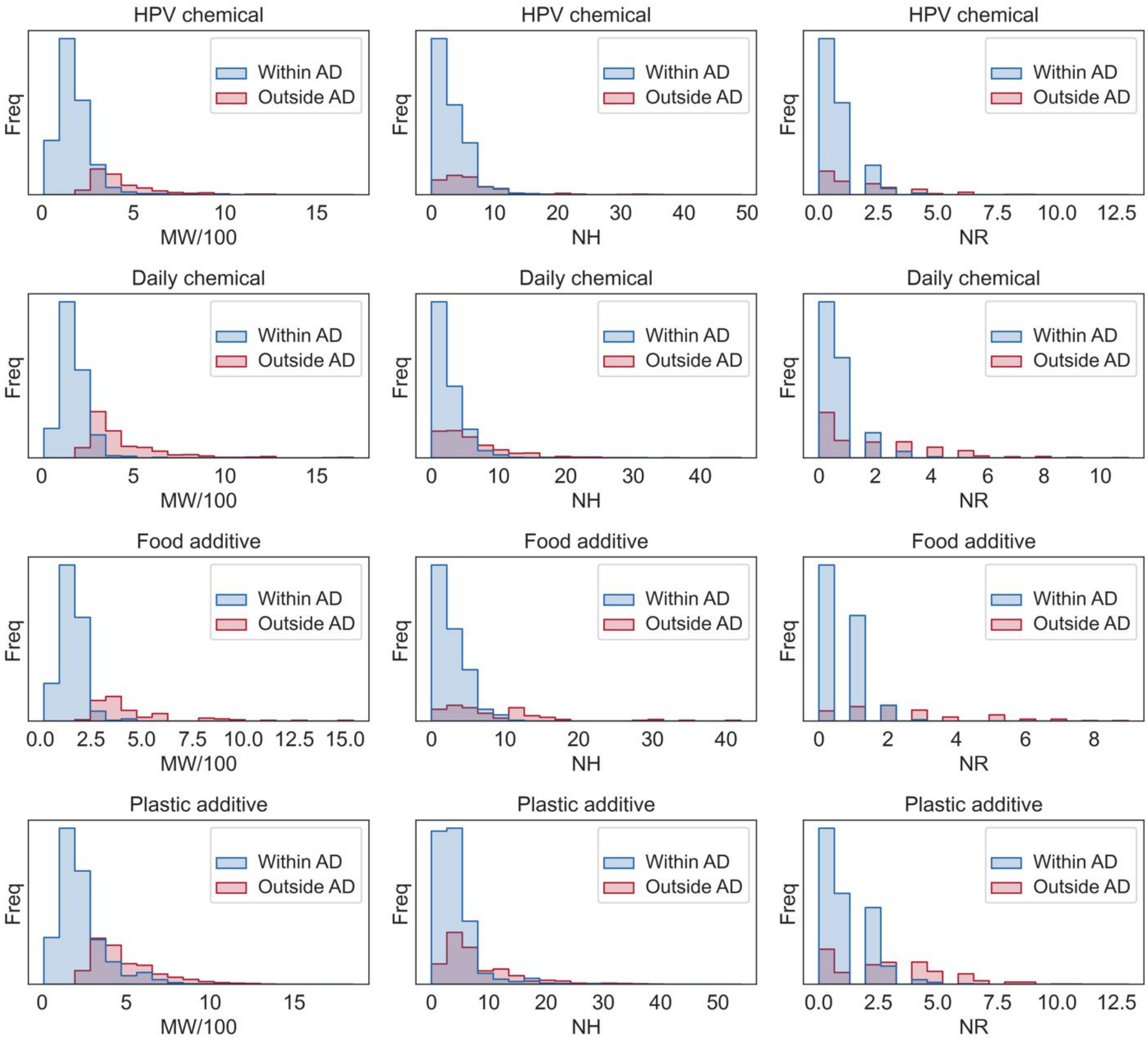

Figure S8. The distribution of molecular weights (MW), number of heteroatoms (NH), and number of rings (NR) of four types of chemicals, including high-production volume (HPV) chemicals (N=2502), daily chemicals (N=1589), plastic additives (N=5281), food additives (N=506), within and outside the applicability domain (AD) of FineChem 2.

References

(1) Wernet, G.; Papadokonstantakis, S.; Hellweg, S.; Hungerbühler, K. Bridging data gaps in environmental assessments: Modeling impacts of fine and basic chemical production. *Green Chemistry* **2009**, *11* (11). DOI: 10.1039/b905558d.

(2) Sun, Y.; Wang, X.; Ren, N.; Liu, Y.; You, S. Improved Machine Learning Models by Data Processing for Predicting Life-Cycle Environmental Impacts of Chemicals. *Environmental Science & Technology* **2022**. DOI: 10.1021/acs.est.2c04945.

(3) Yap, C. W. PaDEL-descriptor: an open source software to calculate molecular descriptors and fingerprints. *Journal of Computational Chemistry* **2011**, *32* (7), 1466-1474. DOI: 10.1002/jcc.21707.

(4) Song, R.; Keller, A. A.; Suh, S. Rapid Life-Cycle Impact Screening Using Artificial Neural Networks. *Environmental Science & Technology* **2017**, *51* (18), 10777-10785. DOI: 10.1021/acs.est.7b02862.
